# First-trimester serum biomarkers in twin pregnancies and adverse obstetric outcomes–a single center cohort study

**DOI:** 10.1007/s00404-024-07547-6

**Published:** 2024-05-12

**Authors:** Alexandra Queirós, Laura Gomes, Inês Pereira, Nádia Charepe, Marta Plancha, Sofia Rodrigues, Álvaro Cohen, Marta Alves, Ana Luísa Papoila, Teresinha Simões

**Affiliations:** 1 Fetal Medicine and Surgery Center, Maternidade Dr. Alfredo da Costa, Unidade Local de Saúde de São José, Lisbon, Portugal; 2 Maternal and Fetal Medicine Unit, Maternidade Dr. Alfredo da Costa, Unidade Local de Saúde de São José, Lisbon, Portugal; 3 Nova Medical School, Universidade Nova de Lisboa, Lisbon, Portugal; 4 Epidemiology and Statistics Unit, Unidade Local de Saúde de São José, Lisbon, Portugal; 5 Centre of Statistics and Its Applications, Universidade de Lisboa, Lisbon, Portugal

**Keywords:** Twin pregnancies, Preterm birth, Fetal growth restriction, Hypertensive disorders, Serum biomarkers, First trimester screening

## Abstract

**Purpose:**

This study aimed to determine the association of first-trimester maternal serum biomarkers with preterm birth (PTB), fetal growth restriction (FGR) and hypertensive disorders of pregnancy (HDP) in twin pregnancies.

**Methods:**

This is a retrospective cohort study of twin pregnancies followed at Maternidade Dr. Alfredo da Costa, Lisbon, Portugal, between January 2010 and December 2022. We included women who completed first-trimester screening in our unit and had ongoing pregnancies with two live fetuses, and delivered after 24 weeks. Maternal characteristics, pregnancy-associated plasma protein-A (PAPP-A) and β-human chorionic gonadotropin (β-hCG) levels were analyzed for different outcomes: small for gestational age (SGA), gestational hypertension (GH), early and late-onset pre-eclampsia (PE), as well as the composite outcome of PTB associated with FGR and/or HDP. Univariable, multivariable logistic regression analyses and receiver-operating characteristic curve were used.

**Results:**

466 twin pregnancies met the inclusion criteria. Overall, 185 (39.7%) pregnancies were affected by SGA < 5th percentile and/or HDP. PAPP-A demonstrated a linear association with gestational age at birth and mean birth weight. PAPP-A proved to be an independent risk factor for SGA and PTB (< 34 and < 36 weeks) related to FGR and/or HDP. None of the women with PAPP-A MoM > 90th percentile developed early-onset PE or PTB < 34 weeks.

**Conclusion:**

A high serum PAPP-A (> 90th percentile) ruled out early-onset PE and PTB < 34 weeks. Unless other major risk factors for hypertensive disorders are present, these women should not be considered candidates for aspirin prophylaxis. Nevertheless, close monitoring of all TwP for adverse obstetric outcomes is still recommended.

## What does this study add to the clinical work

**Table:** 

Higher first-trimester PAPP-A levels, exceeding the 90th percentile, in twin pregnancies, excluded early-onset preeclampsia and preterm birth before 34 weeks. These women should not be considered candidates for aspirin prophylaxis.	

## Introduction

Twin pregnancies (TwP) carry a higher risk of major obstetrical complications, including preterm birth (PTB), fetal growth restriction (FGR), hypertensive disorders of pregnancy (HDP) [1, 2]. PTB is the most prevalent complication in TwP, responsible for significant perinatal morbidity and mortality [1–3]. In singleton pregnancies, maternal serum biomarkers such as PAPP-A (pregnancy-associated plasma protein-A) and β-hCG (β-human chorionic gonadotropin) used for first-trimester screening have been associated with adverse pregnancy outcomes such as pre-eclampsia (PE), FGR, PTB [4–9]. However, for twin pregnancies, comprehensive data on first-trimester biomarkers is lacking and some previous reports have conflicting results [10, 11].

This study aimed to determine the association of first-trimester maternal serum biomarkers with PTB, FGR and HDP in TwP.

## Materials and methods

This is a retrospective analysis of TwP followed at the Maternidade Dr. Alfredo da Costa, Unidade Local de Saúde de São José (ULSSJOSE), Lisbon, Portugal, between January 2010 and December 2022. This is a tertiary perinatal center that cares for the Lisbon area, and serves as a referral center for the South of Portugal. At our center, information about pregnancies and deliveries has been collected prospectively by the Twins Study Group, using an informatic database approved by the Portuguese National Commission for Data Protection. Additional approval for the present study was granted by the Local Ethical Committees of Nova Medical Scholl (No. 81/2020/CEFCM) and ULSSJOSE (No. 950/2020).

Verbal informed consent for anonymous data collection and publication was obtained from all subjects before their inclusion in the Twins Study Group database. Informed written consent was not sought before 2020 for the present study because of the retrospective nature and anonymous data collection. After 2020, written consent was obtained from all subjects for participation and publication.

Routine first-trimester screening for aneuploidies was performed by Fetal Medicine Foundation certified obstetricians. Gestational age was derived from the last menstrual period that was confirmed or corrected by the measurement of fetal crown–rump length of the larger twin in the first trimester scan or from the day of oocyte retrieval in pregnancies after assisted reproductive techniques (ART). Chorionicity was established by ultrasonographic criteria: lambda or T-sign in dichorionic (DC) or monochorionic (MC), respectively, confirmed by careful examination of the delivered placenta by experienced obstetricians and by histopathologic examination.

Maternal blood samples were collected at 10^+0^–13^+6^ weeks into tubes in our laboratory, and serum PAPP-A and β-hCG were obtained from two immunoassay systems: Kryptor (Thermo Fisher Scientific, Clinical Diagnostics, Brahms GmbH, Henningsdorf, Germany) and Cobas (Roche Diagnostics, Basel, Switzerland) and converted to multiples of the median (MoM) using Astraia^®^ software.

The maternal parameters (weight and height, conception method and ethnicity) and medical history (parity, cigarette smoking, chronic hypertension, diabetes mellitus, systemic lupus erythematosus or antiphospholipid syndrome, previous PTB, FGR or PE, family history of PE) were included in the first trimester screening. Inclusion criteria for the study were two live fetuses at the first-trimester scan, and subsequent birth at ≥ 2 weeks gestation. Exclusion criteria were monoamniotic twins, chromosomal and major fetal structural abnormalities, single fetal demise before 24 weeks, abnormal umbilical cord (two vessels or velamentous insertions), TORCH infections, preterm deliveries related to COVID-19 and twin-to twin transfusion syndrome (TTTS) or twin anemia polycythemia sequence (TAPS) and lost to follow-up.

Obstetric interventions were done according to the institutional clinical guidelines in an individualized practice and no randomization was instituted. In normally progressing gestations, we offered elective termination of pregnancy at 36–38 completed weeks of gestation and iatrogenic preterm deliveries were carried out based on maternal and/or fetal conditions. HDP were defined according to the International Society for the Study of Hypertension in Pregnancy (ISSHP) classification, diagnosis, and management recommendations for international practice [12]. FGR was defined according to “Consensus definition” in TwP described by Khalil A et al. [13].

SGA was defined as birth weight falling below the 10th, 5th, or 3rd percentile for each gestational age. To establish our own population percentiles (unpublished data), we analyzed all twins followed in our institution who had uncomplicated pregnancies (excluding maternal, obstetric and fetal diseases) and delivered two live newborns after 36 weeks between the years 1994 and 2002. Our population percentiles for each gestational age were adjusted for chorionicity and based on ultrasound fetal weight estimations between 20 and 35 weeks, as well as birthweight data after 36 weeks, excluding birthweights of twins born prematurely under 36 weeks.

The evaluated outcomes included SGA in one or both twins, GH, early and late-onset PE (< 34 and ≥ 34 weeks), and PTB (< 32, < 34 and < 36 weeks), as well as the composite outcome of PTB (< 32, < 34 and < 36 weeks) associated with FGR and/or HDP.

## Statistical analysis

An exploratory analysis of the variables under study was carried out with categorical variables being described as frequencies (percentages), and the remaining variables as mean (standard deviation). Normal distribution of the quantitative variables was verified using the Shapiro–Wilk test. Outcome variables were associated with all clinical and demographic variables using Mann–Whitney, Chi-square, or Fisher’s exact tests, as appropriate. The sensitivity, false positive rate, specificity, and positive and negative likelihood ratios for PAPPA-A serum biomarker with the cut-off points at 10th percentile, 1.0 MoM and 90th percentile were estimated. To study the association between the outcomes with maternal/ pregnancy characteristics and serum biomarkers, logistic regression models were used. For the multivariable models, all the variables that in the univariable analysis attained a *p*-value ≤ 0.25 were selected. Adjusted odds ratios (OR) were estimated with corresponding 95% confidence intervals (95% CI). Discriminative ability and calibration of these models were assessed by the area under the receiver-operating characteristic curve (AUC) and the Hosmer–Lemeshow (HL) goodness-of-fit test, respectively.

Although a significance level of α = 0.05 was considered, several variables of clinical relevance were maintained in the final models despite having a *p*-value > 0.05. Data analysis was performed using the statistical package for the social sciences for windows (IBM Corp. released 2021. version 28.0).

## Results

**Fig. 1:**
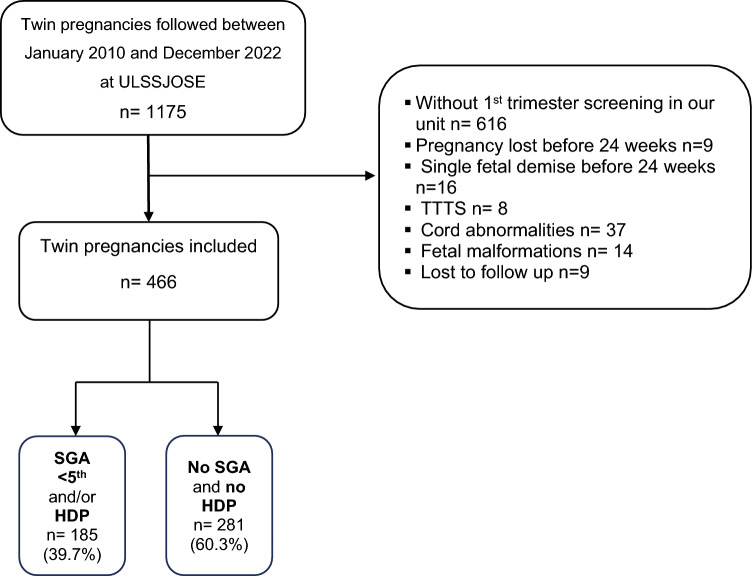
Flowchart showing inclusion pregnancies who underwent first-trimester screening at ULSSJOSE. ULSSJOSE - Unidade Local de Saúde de São José, *HDP* hypertensive disorders of pregnancy, *SGA* small to gestational age, *TTTS* twin to twin transfusion syndrome

Of the 1175 TwP followed between January 2010 and December 2022 at ULSSJOSE, the dataset included 466 TwP that met the inclusion criteria: 384 (82.4%) dichorionic and 82 (17.6%) monochorionic (Fig. 1). Of these, 308 (66.1%) were nulliparous, and 22 (4.7%) had chronic hypertension (CH). Maternal and pregnancy characteristics are summarized in Table 1. Among women without prior chronic hypertension, 71 (15.2%) developed GH, while in women with pre-existing chronic hypertension (CH), seven experienced an exacerbation of the condition, and 33 (7.1%) women progressed to preeclampsia (PE). Two single fetal demise occurred in two dichorionic pregnancies, one at 25 and another at 36 weeks. Pregnancies affected with one or both infants classified as SGA under the 3rd, 5th, and 10th percentiles were 97 (20.8%), 126 (27.0%), and 153 (32.8%), respectively. PTB associated with HDP and/or FGR occurred in 46 cases (9.9%) before 34 weeks and 95 cases (20.4%) before 36 weeks. Overall, 185 (39.7%) pregnancies were affected by SGA < 5th percentile and/or HDP.

**Table 1:** Demographic, pregnancy characteristics and serum biomarkers (PAPP-A and β-hCG) in 466 twin pregnancies who underwent 1st trimester screening

*n* = 466	Mean (SD) or *n* (%)
**Maternal age (years)**	32.9 (4.9)
Age ≥ 40 years	30 (6.4%)
**BMI** (Kg/m^2^)	24.5 (4.3)
BMI < 20	57 (12.2%)
BMI ≥ 20 < 35	241 (51.7%)
BMI ≥ 25 < 30	114 (24.5%)
BMI ≥ 30	54 (11.6%)
**Ethnicity**	
Caucasian	399 (85.6%)
African	52 (11.1%)
East Asian	14 (2.1%)
Mixed/others	1 (0.2%)
**Parity**	
Nulliparous	308 (66.1%)
Parous with prior PTB	17 (10.7%)
Parous with prior PE	6 (3.7%)
Parous with prior SGA	11 (6.9%)
**Method of conception**	
Spontaneous	260 (55.8%)
Ovulation inductions	16 (3.4%)
ART	190 (40.8%)
**Chorionicity**	
Monochorionic diam	82 (17.6%)
Dichorionic	384 (82.4%)
**Smoker**	49 (10.5%)
**Chronic hypertension**	22 (4.7%)
**SLE/APS/thrombophilia**	14 (3.0%)
**Diabetes mellitus**	3 (0.6%)
**Aspirin prophylactic intake (started < 20 weeks)**	60 (12.6%)
**1st trimester Mean Arterial Pressure**	85.6 (8.5)
**PAPP_A MoM**	1.1 (0.6)
**β-hCG MoM**	1.1 (0.7)

*β-hCG* β-human chorionic gonadotropin, *BMI* body mass index, *PAPP-A* pregnancy-associated plasma protein-A, *PTB* preterm birth, *PE* preeclampsia, *SGA* small to gestational age, *SD* standard deviation, *ART* artificial reproductive techniques, *SLE* systemic lupus erythematosus, *APS* antiphospholipid syndrome

In univariable analysis of maternal/pregnancy characteristics, MC pregnancies exhibited increased odds of PTB < 36 weeks associated with FGR and/or HDP compared to DC: 32.0% vs 18.9%, OR 2.0 (95% CI 1.3–3.2, *p* = 0.002). Additionally, MC pregnancies showed higher odds of early onset PE < 34 weeks, although without reaching statistical significance: 3.3% vs 0.9%, OR 3.7 (95% CI 0.8–16.9, *p* = 0.068).

Nulliparous, older women (≥ 40 years old), and pregnancies resulting from ART demonstrated increased odds of GH: 18.2% vs 8.0%, OR 2.5 (95% CI 1.4–4.6, *p* = 0.001); 25.7% vs 13.9%, OR 2.1 (95% CI 1.0–4.8, *p* = 0.049) and 20.2% vs 11.2%, OR 2.0 (95% CI 1.2–3.2, *p* = 0.004), respectively. ART pregnancies also showed increased odds of late-onset pre-eclampsia: 7.3% vs 3.3%, OR 2.3 (95% CI 1.0–5.0, *p* = 0.030).

Women with low body mass index (BMI) (< 20 kg/m^2^) showed increased odds of SGA < 3rd and 5th percentiles, 31.1% vs 19.7% OR 1.8 (95% CI 1.0–3.1, *p* = 0.025), and 41.9% vs 25.9%, OR 2.1 (95% CI 1.2–3.4, *p* = 0.003), respectively. Low maternal BMI was also associated with higher odds of PTB < 36 weeks related with FGR and/or HDP, 33.8% vs 19.9%, OR 2.0 (95% CI 1.2–3.4, *p* = 0.007). Maternal obesity was associated with increased of PTB < 34 weeks related with FGR and/or HDP, 19.4% vs 8.2%, OR 2.6 (95% CI 1.3–5.4, *p* = 0.005), and late-onset PE, 9.7% vs 4.1% OR 2.4 (95%CI 0.9–6.4, *p* = 0.061), although the latter did not reach statistical significance.

Out of the 60 patients under aspirin prophylaxis administered before 20 weeks, two (3.3%) developed early-onset PE, and 10 (16.7%) had PTB < 34 weeks related to FGR and/or HDP. In contrast, within the group without instituted prophylaxis, early PE occurred in six (1.5%) patients, and 36 (8.6%) had PTB < 34 weeks related to FGR and/or HDP, with OR of 2.2 (95% CI 0.4–11.6, *p* = 0.302), and 2.0 (95% CI 0.9–4.3, *p* = 0.059), respectively. In both cases, *p*-value did not reach statistical significance.

**Fig. 2:**
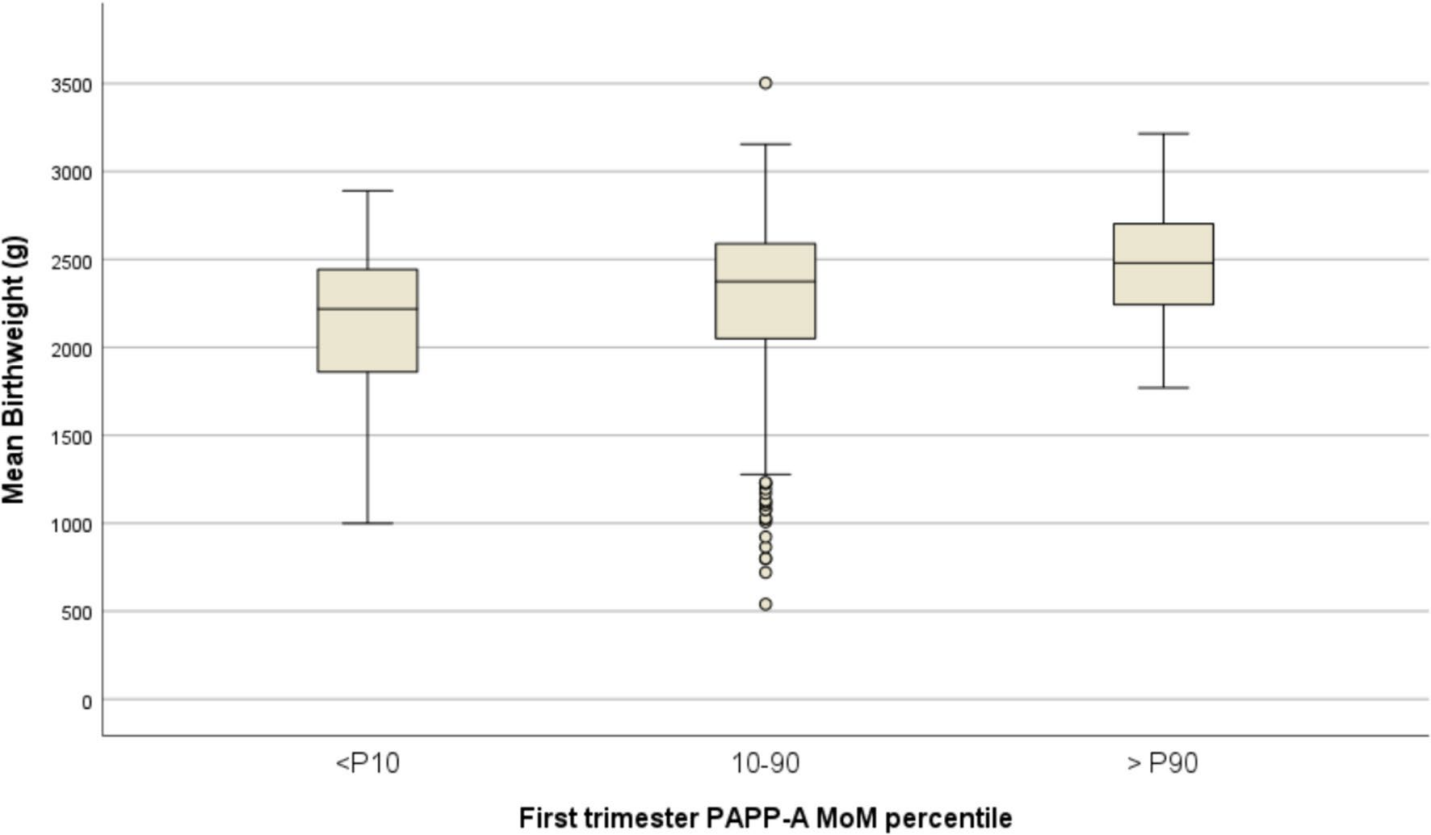
First trimester serum pregnancy-associated plasma protein-A (PAPP-A) in twin pregnancies and mean birthweight (circles represent moderate outliers)

**Fig. 3:**
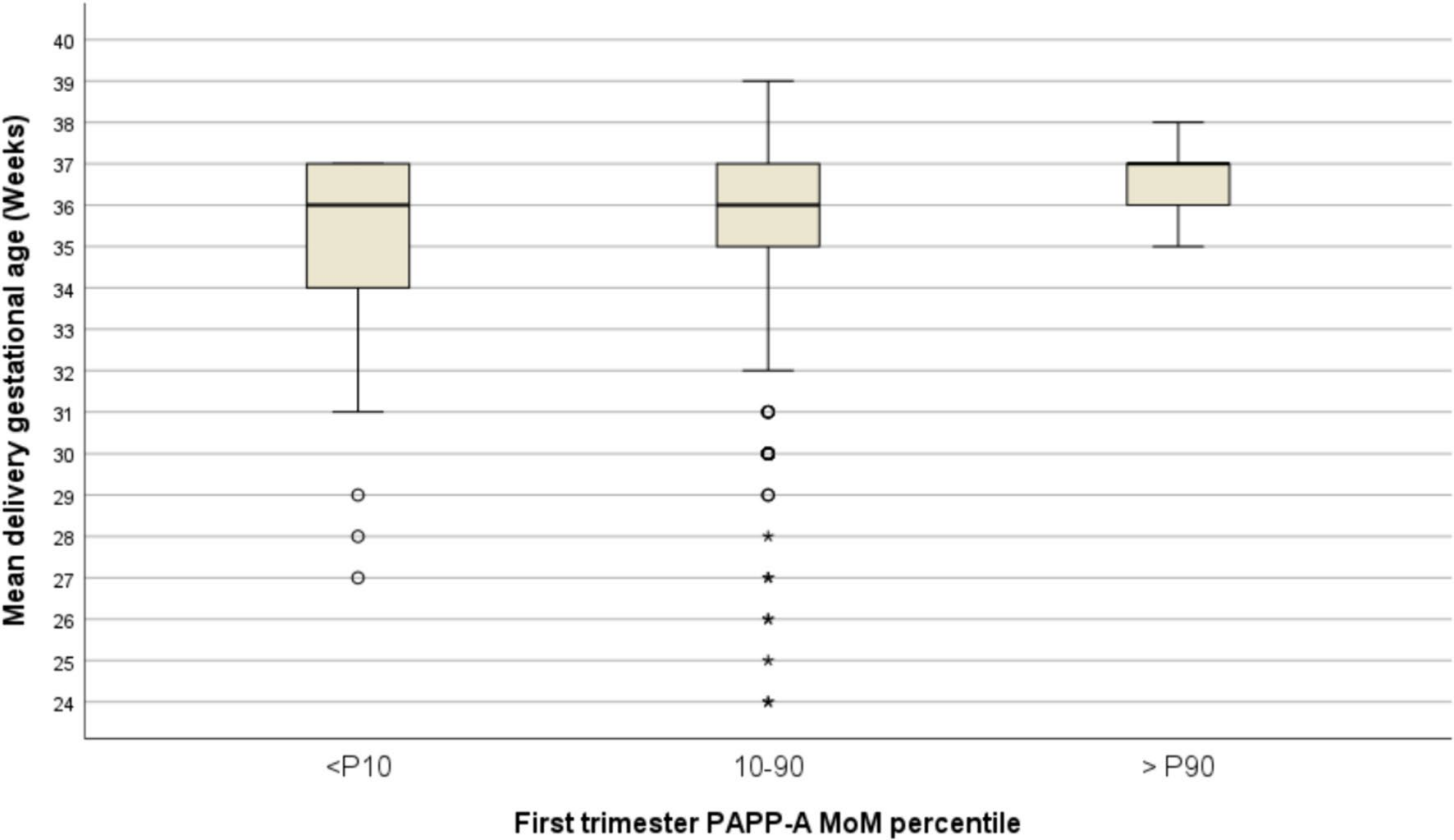
First trimester serum pregnancy-associated plasma protein-A (PAPP-A) in twin pregnancies and mean gestational age (circles represent moderate outliers and asterisks extreme outliers)

PAPP-A demonstrated a linear association with gestation age at birth and mean birth weight, *p* = 0.003 and *p* < 0.001, respectively (Table 2 and Fig. 2). Additionally, low levels of serum biomarker PAPP-A, specifically < 0.50 MoM corresponding to the 10th percentile in our dataset, were associated with increased odds of SGA < 3rd percentile, OR 2.1 (95% CI: 1.1–4.2, *p* = 0.016), and PTB < 32 and < 36 weeks associated with FGR and/or HDP, OR 3.3 (95% CI 1.2–8.9, *p* = 0.023), and OR 2.0 (95% CI 1.0–3.9, *p* = 0.030), respectively. Despite these findings in univariable analyses, the use of PAPP-A < 10th percentile alone for predicting adverse outcomes is poor. The best detection rate (25%) was observed for PTB < 32 weeks, but it came with the highest false positive rate (87.5%). Even when considering a higher cut-off of 1.0 MoM, the sensitivity improved to 75%, albeit with a high false positive rate of 92.2% (Table 3). On the other hand, none of the women with high level of PAPP-A, over the 90th percentile corresponding to 1.82 MoM, developed early-onset PE, or PTB < 32 or < 34 weeks, whether spontaneous or iatrogenic (Fig. 3).

**Table 2:** Univariable analyses of first trimester maternal serum PAPP-A and obstetric outcomes: PTB, SGA/FGR and HDP in twin pregnancies

	Total *n* = 466 mean (SD) or *n* (%)	PAPP-A MoM^a^	PAPP-A MoM < 10th (0.50)		PAPP-A MoM > 90th (1.82)	
		Statistics B or OR (95 CI) *p*-value	Mean (SD) or *n* (%)	Statistics B or OR (95% CI) *p*-value	Mean (SD) or n(%)	Statistics B or OR (95% CI) *p*-value
Gestational age at delivery (weeks)	35.4 (2.3)	0.26 (0.1–0.4) 0.003	34.7 (2.6)	− 0.8 (− 1.5– − 0.1) < 0.001	36.3 (0.8)	1.0 (0.3–1.7) < 0.001
Mean birthweight (g)	2278 (482)	73 (38.9–104.1) < 0.001	2074 (508)	− 266 (− 372– − 0.8) 0.002	2487 (343)	232 (86–378) < 0.001
One or both SGA < 3rd percentile	97 (21.0%)	0.7 (0.6–0.9) 0.007	16 (34.8%)	2.1 (1.1–4.2) 0.016	7 (15.2%)	0.6 (0.2–1.4) 0.307
One or both SGA < 5th percentile	126 (27.3%)	0.8 (0.6–0.9) 0.007	18 (39.1%)	1.8 (0.9–3.4) 0.058	7 (15.2%)	0.4 (0.2–1.0) 0.052
One or both SGA < 10th percentile	153 (33.2%)	0.8 (0.6–0.9) 0.016	20 (43.5%)	1.6 (0.8–3.0) 0.118	9 (19.6%)	0.5 (0.2–0.9) 0.039
Early-onset PE < 34 weeks	8 (1.7%)	0.6 (0.2–1.3) 0.187	0 (0%)	- 1.000	0 (0%)	– 1.000
Late-onset PE ≥ 34 weeks	25 (5.4%)	1.0 (0.7–1.4) 0.928	3 (6.5%)	1.2 (0.3–4.3) 0.727	2 (4.3%)	0.7 (0.2–3.4) 1.000
Gestational hypertension^b^	77 (16.5%)	0.7 (0.4–1.1) 0.188	8 (17.4%)	1.1 (0.5–2.4) 0.867	5 (10.9%)	0.6 (0.2–1.6) 0.277
PTB < 32 weeks with FGR and/or GH	24 (5.2%)	0.5 (0.3–0.9) 0.021	6 (13%)	3.3 (1.2–8.9) 0.023	0 (0%)	– 0.153
PTB < 34 weeks with FGR and/or GH	46 (9.9%)	0.6 (0.4–0.9) 0.009	6 (13%)	1.4 (0.5–3.5) 0.435	0 (0%)	– 0.015
PTB < 36 weeks with FGR and/or GH	95 (20.4%)	0.7 (0.6–0.9) 0.021	15 (32.6%)	2.0 (1.0–3.9) 0.030	4(8.7%)	0.3 (0.1–0.9) 0.038
PTB < 32 weeks (all cases)	35 (7.5%)	0.6 (0.4–0.9) 0.010	8 (17.4%)	3.0 (1.3–7.2) 0.015	0 (0%)	– 0.037
PTB < 34 weeks (all cases)	70 (15.0%)	0.6 (0.4–0.8) < 0.001	10 (21.7%)	1.6 (0.7–3.5) 0.179	0(0%)	– < 0.001
PTB < 36 weeks (all cases)	156 (33.5%)	0.8 (0.7–0.9) 0.026	22 (47.8%)	1.9 (1.0–3.6) 0.030	9 (19.6%)	0.4 (0.2–0.9) 0.035

^a^PAPP-A as a continuous variable, with beta coefficient estimate (B) and Odds Ratios estimates (OR) representing the increase for each 0.5 MoM increment in PAPP-A

^b^Including 71 cases of gestational hypertension (new onset) and 6 cases of exacerbation of chronic hypertension

*FGR* fetal growth restriction, *GH* gestational hypertension, *HDP* hypertensive disorders of pregnancy, *MoM* multiple of the median, *PAPP-A* pregnancy-associated plasma protein-A, *PE* preeclampsia, *PTB* preterm birth, *SD* standard deviation, *SGA* small to gestational age

**Table 3:** Performance of different cut-off of PAPPA-A levels in predicting adverse outcomes: SGA/FGR and HDP related with PTB in twin pregnancies

	Sensitivity (%)	False positive rate (%)	Specificity (%)	Positive likelihood	Negative likelihood
**Low PAPPA-A < 0.5 MoM (P10th)**					
One or both SGA < 3rd percentile	16.5	65.2	91.8	2.00	0.91
One or both SGA < 5th percentile	14.3	60.9	91.6	1.71	0.94
One or both SGA < 10th percentile	15.0	56.5	91.6	1.78	0.93
PTB < 32 weeks with FGR/HDP	25.0	87.0	91.0	2.76	0.82
PTB < 34 weeks with FGR/HDP	13.0	87.0	90.5	1.37	0.96
PTB < 36 weeks with FGR/HDP	15.8	67.4	91.6	1.89	0.92
**Low PAPPA-A < 1.0 MoM**					
One or both SGA < 3rd percentile	60.8	74.2	53.3	1.30	0.74
One or both SGA < 5th percentile	57.1	68.6	53.1	1.22	0.81
One or both SGA < 10th percentile	54.2	63.8	52.6	1.14	0.87
PTB < 32 weeks with FGR/HDP	75.0	92.2	52.0	1.56	0.48
PTB < 34 weeks with FGR/HDP	58.7	88.3	51.7	1.21	0.80
PTB < 36 weeks with FGR/HDP	56.8	76.5	52.6	1.20	0.82
**PAPPA-A < 1.82 MoM (P90th)**					
One or both SGA < 3rd percentile	92.8	78.3	10.7	1.04	0.67
One or both SGA < 5th percentile	94.4	71.3	11.6	1.07	0.48
One or both SGA < 10th percentile	94.1	65.3	12.0	1.07	0.49
PTB < 32 weeks with FGR/HDP	100.0	91.7	10.7	1.12	0.00
PTB < 34 weeks with FGR/HDP	100.0	83.3	11.6	1.13	0.00
PTB < 36 weeks with FGR/HDP	94.2	65.0	11.9	1.07	0.48

*FGR* fetal growth restriction, *GH* gestational hypertension, *HDP* hypertensive disorders of pregnancy, *MoM* multiple of the median, *PAPP-A* pregnancy-associated plasma protein-A, *PE* preeclampsia, *PTB* preterm birth, *SD* standard deviation, *SGA* small to gestational age

In our cohort, there was no association between β-hCG levels and any of the evaluated outcomes.

In the multivariable logistic regression analysis for the association study with different outcomes, PAPP-A proved to be an independent risk factor for SGA, and PTB < 34 and < 36 weeks associated with FGR and/or HDP (Table 4).

**Table 4:** Multivariable regression analyses for SGA, HDP and PTB in twin pregnancies

Independent variables	Adjusted OR (95% CI)	*p*-value
**One or both SGA < 3rd percentile**		
PAPP-A MoM	0.53 (0.34–0.84)	0.007
BMI < 20 (Kg/m^2^)	1.68 (0.90–3.15)	0.101
**One or both SGA < 5th percentile**		
PAPP-A MoM	0.53 (0.35–0.80)	0.003
BMI < 20 (Kg/m^2^)	1.98 (1.10–3.57)	0.023
Nulliparas	1.51 (0.95–2.40)	0.081
Chronic hypertension	2.21(0.89–5.50)	0.086
Maternal age ≥ 40 years	1.93 (0.87–4.33)	0.107
**One or both SGA < 10th percentile**		
PAPP-A MoM	0.63 (0.43–0.91)	0.003
BMI < 20 (Kg/m^2^)	2.02(1.14–3.56)	0.015
Nulliparas	1.66 (1.07–2.57)	0.022
**One or both SGA < 5th percentile and PTB < 34 weeks**		
PAPP-A MoM	0.39 (0.18–0.85)	0.018
BMI ≥ 30 (Kg/m^2^)	2.08 (0.89–4.86)	0.089
**One or both SGA < 5th percentile and PTB < 36 weeks**		
PAPP-A MoM	0.47 (0.28–0.81)	0.006
Monochorionic	1.92 (1.05–3.50)	0.033
BMI < 20 (Kg/m^2^)	1.98 (1.01–3.88)	0.045
Chronic hypertension	2.96 (1.07–8.16)	0.035
**All gestational hypertension (new onset, chronic hypertension excluded)**		
Mean arterial pressure	1.07 (1.02–1.12)	0.002
BMI ≥ 30 (Kg/m^2^)	2.2 (0.87–5.67)	0.095
Nulliparas	1.7 (0.8–3.58)	0.163
**Gestational hypertension (new onset, chronic hypertension excluded) and PTB < 36 weeks**		
Nulliparas	2.85 (1.14–7.09)	0.024
BMI ≥ 30 (Kg/m^2^)	2.59 (1.00–6.70)	0.050
Late onset preeclampsia (≥ 34 weeks)		
Mean arterial pressure	1.05 (1.00–1.11)	0.047
BMI ≥ 30 (Kg/m^2^)	2.56 (0.84–7.72)	0.095
**PTB < 34 weeks with FGR and/or HDP**		
PAPP-A MoM	0.41(0.20–0.82)	0.013
BMI ≥ 30 (Kg/m^2^)	2.61 (1.22–6.57)	0.013
**PTB < 36 weeks with FGR and/or HDP**		
PAPP-A MoM	0.58 (0.37–0.92)	0.020
BMI < 20 (Kg/m^2^)	1.92 (1.03–3.60)	0.039
Chronic hypertension	2.52 (0.97–6.57)	0.057
Monochorionic	1.67 (0.95–2.93)	0.073
**All cases with SGA < 5th percentile and/or HDP**		
PAPP-A MoM	0.59 (0.43–0.84)	0.004
Nulliparas	1.73 (1.14–2.63)	0.010
Chronic hypertension	3.22 (1.27–8.16)	0.013
BMI < 20 (Kg/m^2^)	1.98 (1.12–3.52)	0.019
Maternal age ≥ 40 years	2.55 (1.16–5.58)	0.019

*BMI* body mass index, *FGR* fetal growth restriction, *GH* gestational hypertension, *HDP* hypertensive disorders of pregnancy, *MoM* multiple of the median, *OR* odds ratio, *PAPP-A* pregnancy-associated plasma protein-A, *PTB* preterm birth, *SGA* small to gestational age

Extreme maternal BMI values (< 20 and ≥ 30 kg/m^2^) independently contributed to adverse outcomes, including SGA in low BMI and PTB < 34 weeks with FGR and/or HDP and GH related with PTB < 36 weeks. Additionally, elevated mean arterial pressure in the first-trimester increased the odds of GH and late-onset PE. Due to the low incidence of early-onset preeclampsia, we were unable to find a well-fitted model for this outcome.

The regression models, which incorporated maternal factors and first-trimester PAPP-A MoM measurements, demonstrated superior performance compared to models based solely on maternal factors. Some associations identified in the univariable analysis, such as the ART mode of conception, lost statistical significance in the multivariable analysis. The obtained AUCs revealed limited discriminative performance of the multivariable models (ranging between 0.60 and 0.70) and a good calibration, assessed by the HL test (Table 5).

**Table 5:** Performance of multivariable logistic regression models using maternal factors and PAPP-A MoM for predicting SGA, HDP and PTB in twin pregnancies

Outcome	Hosmer–Lemeshow test significance	AUC (95% CI)
One or both SGA < 3rd perc	0.207	0.601 (0.536–0.667)
One or both SGA < 5th perc	0.404	0.638 (0.582–0.694)
One or both SGA < 10th perc	0.201	0.619 (0.565–0.673)
One or both SGA < 5th perc. and PTB < 34 weeks	0.531	0.646 (0566–0.726)
One or both SGA < 5th perc. and PTB < 36 weeks	0.319	0.660 (0.592–0.728)
All gestational hypertension	0.641	0.708 (0.635–0.782)
GH and PTB < 36 weeks	0.859	0.579 (0.457–0.702)
Late onset PE	0.306	0.708 (0.616–0.802)
PTB < 34 weeks with FGR and/or HDP	0.408	0.652 (0.572–0.729)
PTB < 36 weeks with FGR and/or HDP	0.445	0.642 (0.579–0.705)
All cases with SGA < 5th percentile and/or HDP	0.129	0.655 (0.604–0.705)

*AUC* area under the receiver–operating characteristic curve, *FGR* fetal growth restriction, *GH* gestational hypertension, *HDP* hypertensive disorders of pregnancy, MoM multiple of the median, *PAPP-A* pregnancy-associated plasma protein-A, *PE* preeclampsia, *PTB* preterm birth, *SGA* small to gestational age

## Discussion

In the era of non-invasive prenatal testing (NIPT), the importance of first-trimester screening, which combines ultrasound with biochemical and biophysical markers, remains a paramount in prenatal care. In singletons, if NIPT is used as a second-line approach for women with intermediate risk, it has been proved to be an efficient and cost-effective approach for detecting major trisomies and identifying more fetuses with potential abnormalities [14]. If we employ this strategy, along with screening for aneuploidies, first-trimester combined screening offers the benefits of identifying pregnancies at increased risk of PE, spontaneous PTB, and FGR, although the latter exhibits poorer performance [9, 15, 16]. The same first-trimester screening approach can be extended to TwP, although with less conclusive evidence regarding its effectiveness [17].

TwP carries a heightened risk of obstetric conditions, including FGR and HDP. Often, especially when diagnosed before term, these two conditions are associated and are part of manifestations related to poor placental perfusion. In our study, in 66.7% of cases of early-onset PE, it was associated with SGA < 5th percentile. Conversely 21.1% of women with SGA < 5th percentile and PTB < 34 weeks developed GH. Proctor et al. found these same associations in TwP when birthweight charts adjusted for twins were used in the data analysis [18].

PE is a complex condition caused by various factors, processes, and pathways [19]. Some maternal and pregnancy conditions are recognized as risk factors for poor obstetric outcomes. For example, extreme values of maternal BMI are associated with higher odds of adverse maternal and fetal/neonatal outcomes, as well as higher rates of placental maternal vascular malperfusion [20, 21]. Additionally, ART was found to be an independent risk factor for preterm delivery in twin pregnancies with preeclampsia [22]. Fetal sex can also play a role in modifying the odds of PTB or HDP in twin pregnancies, as demonstrated in previous studies [23, 24]. However, in our small cohort, only extremes BMI values achieved statistical significance for some outcomes in the multivariable analysis.

PAPP-A plays a crucial role in pregnancy by regulating placental function, fetal growth, and placental development. Its biological mechanism involves the modulation of insulin-like growth factor activity, which influences various aspects of pregnancy and fetal development. Maternal concentrations reflect the placental volume and likely the amount of trophoblastic tissue [25]. There is evidence suggesting that disorders in deep placentation and failure in the physiological transformation of spiral arteries, leading to placental dysfunction, are also associated with spontaneous PTB in a substantial portion of cases [26].

Although in this study, women with low PAPP-A showed an increased risk for SGA and PTB < 32 and < 36 weeks, related to FGR and/or HDP, this marker alone has low predictive value to detect the majority of cases at risk. Our findings align with the conclusions of a systematic review and meta-analysis derived from singletons [8]. Conversely, high measurement of this biomarker > 90th percentile excluded all cases of PTB < 34 weeks, whether spontaneous or iatrogenic. This may reflect a larger placental mass and improved trophoblastic function in those pregnancies, facilitating adequate fetal growth and prolonging gestation. Higher levels of PAPP-A can be very reassuring for women and clinicians, but they are applicable to only 10% of our population and this finding should be confirmed in larger datasets. Nevertheless, clinicians and patients may question the use of cut-off values of a single biomarker to define the clinical risk in TwP.

Besides β-hCG and PAPP-A, there are many other candidate placental products to be employed as surrogates of placental function. A systematic review of early pregnancy biomarkers in PE found low predictive values using individual biomarkers which included a disintegrin and metalloprotease 12 (ADAM-12), inhibin-A, PAPP-A, placental growth factor (PlGF) and placental protein 13 (PP-13) [27]. PLGF and PAPP-A are the two more commonly used for the fetal medicine foundation first-trimester screening but should be combined with maternal factors, MAP, and uterine doppler for better performance.

Unfortunately, we do not routinely measure PLGF in the first trimester, and we do not have a biobank with serological samples to carry out this measurement. Another limitation of our study is the fact that many pregnancies are only followed at our institution after 14 weeks, which makes it impossible to carry out first-trimester screening.

In clinical practice, screening tests are useful for risk stratification and prevention measures. In the case of singleton pregnancies, the use of aspirin for the prevention of preterm PE in high-risk patients is well established [28]. The reduction in the risk of early-onset PE is accompanied by a decrease in the risk of PTB and FGR. In the ASPRE trial, use of aspirin reduced the overall incidence of SGA < 10th percentile by about 40% in newborns at < 37 weeks’ gestation and by about 70% in newborns at < 32 weeks [29].

The American College of Obstetricians and Gynecologists (ACOG) recommends the use of aspirin in all TwP due to the higher risk for hypertensive disorders [30]. In contrast, the National Institute for Health and Care Excellence (NICE) guidelines suggest aspirin if two moderate risks are present; for example, all nulliparous women with a twin gestation should take aspirin [31]. In the latter scenario, about 73% (all nulliparas and multiparas with risk factors) of our patients would be prescribed medication. In our practice, we opted to prescribe aspirin exclusively to women with significant risk conditions. This approach resulted in a lower rate of selection (12.6%) for women under prophylaxis. We cannot precisely estimate the potential reduction in the occurrence of adverse outcomes with this option in our population but in our cohort, the vast majority of women who had PTB < 34 weeks related to FGR and/or HDP were not under aspirin prophylaxis. Would the outcomes be different if a different approach were implemented?

Attempts to clarify if aspirin intake in twins is beneficial have been made in the past [32, 33]. A systematic review and meta-analysis showed that the administration of aspirin in women with TwP reduced the risk of PE but not FGR. The overall quality of evidence is low and this highlighted the need for randomized controlled trials elucidating the actual role of aspirin in affecting maternal and perinatal outcomes in TwP [34].

## Conclusions

While clinicians await better evidence and updated guidelines regarding the use of aspirin in twins, decisions in the field must be made considering the risks and benefits of instituting drug prophylaxis. Isolated low levels of PAPP-A can be considered but with limited predictive value for adverse outcomes. On the other hand, this study demonstrated that a high serum PAPP-A, exceeding the 90th percentile, ruled out early-onset PE and PTB before 34 weeks. This finding should be validated with larger datasets. Unless other major risk factors for hypertensive disorders are present, these women should not be considered candidates for aspirin prophylaxis. Nevertheless, close monitoring of all TwP for adverse obstetric outcomes is still recommended.

## Data Availability

We would like to disclose that while the data underlying our study are not publicly available due to data protection reasons, they can be made available for scientific purposes upon request. Interested parties may contact the corresponding author, Alexandra Queirós, at alexandra.queiros@ulssjose.min-saude.pt or the investigation department at projetos.inv@ulssjose.min-saude.pt to inquire about accessing the data for scientific research.

## References

[1] Santolaya J (2012). Twins-twice more trouble?. Clin Obstet Gynecol.

[2] Wainstock T, Yoles I, Sergienko R, Sheiner E (2023). Twins vs singletons-long-term health outcomes. Acta Obstet Gynecol Scand.

[3] Chauhan SP, Scardo JA, Hayes E, Abuhamad AZ, Berghella V (2010). Twins: prevalence, problems, and preterm births. YMOB.

[4] Beta J, Akolekar R, Ventura W, Syngelaki A, Nicolaides KH (2011). Prediction of spontaneous preterm delivery from maternal factors, obstetric history and placental perfusion and function at 11–13 weeks. Prenat Diagn.

[5] Wright A, Guerra L, Pellegrino M, Wright D, Nicolaides KH (2016). Maternal serum PAPP-A and free β-hCG at 12, 22 and 32 weeks’ gestation in screening for pre-eclampsia. Ultrasound Obstet Gynecol.

[6] Giorgione V, Quintero Mendez O, Pinas A, Ansley W, Thilaganathan B (2022). Routine first-trimester pre-eclampsia screening and risk of preterm birth. Ultrasound Obstet Gynecol.

[7] Chiu CPH, Feng Q, Chaemsaithong P (2022). Prediction of spontaneous preterm birth and preterm prelabor rupture of membranes using maternal factors, obstetric history and biomarkers of placental function at 11–13 weeks. Ultrasound Obstet Gynecol.

[8] Morris RK, Bilagi A, Devani P, Kilby MD (2017). Association of serum PAPP-A levels in first trimester with small for gestational age and adverse pregnancy outcomes: systematic review and meta-analysis. Prenat Diagn.

[9] Cavoretto PI, Farina A, Salmeri N, Syngelaki A, Tan MY, Nicolaides KH (2024). First trimester risk of preeclampsia and rate of spontaneous birth in patients without preeclampsia. Am J Obstet Gynecol.

[10] Svirsky R, Levinsohn-Tavor O, Feldman N, Klog E, Cuckle H, Maymon R (2016). First- and second-trimester maternal serum markers of pre-eclampsia in twin pregnancy. Ultrasound Obstet Gynecol.

[11] Saletra-Bielińska A, Kosińska-Kaczyńska K, Szymusik I (2020). Both low and high PAPP-A concentrations in the first trimester of pregnancy are associated with increased risk of delivery before 32 weeks in twin gestation. J Clin Med.

[12] Brown MA, Magee LA, Kenny LC (2018). Hypertensive disorders of pregnancy: ISSHP classification, diagnosis, and management recommendations for international practice. Hypertension.

[13] Khalil A, Beune I, Hecher K (2019). Consensus definition and essential reporting parameters of selective fetal growth restriction in twin pregnancy: a delphi procedure. Ultrasound Obstet Gynecol.

[14] Ye C, Duan H, Liu M, Liu J, Xiang J, Yin Y, Zhou Q, Yang D, Yan R, Li R (2023). The value of combined detailed first-trimester ultrasound-biochemical analysis for screening fetal aneuploidy in the era of non-invasive prenatal testing. Arch Gynecol Obstet.

[15] Papastefanou I, Wright D, Syngelaki A, Souretis K, Chrysanthopoulou E, Nicolaides KH (2021). Competing-risks model for prediction of small-for-gestational-age neonate from biophysical and biochemical markers at 11–13 weeks’ gestation. Ultrasound Obstet Gynecol.

[16] O'Gorman N, Wright D, Syngelaki A, Akolekar R, Wright A, Poon LC, Nicolaides KH (2016). Competing risks model in screening for preeclampsia by maternal factors and biomarkers at 11–13 weeks gestation. Am J Obstet Gynecol.

[17] Benkő Z, Wright A, Rehal A (2021). Prediction of pre-eclampsia in twin pregnancy by maternal factors and biomarkers at 11–13 weeks’ gestation: data from EVENTS trial. Ultrasound Obstet Gynecol.

[18] Proctor LK, Kfouri J, Hiersch L (2019). Association between hypertensive disorders and fetal growth restriction in twin compared with singleton gestations. Am J Obstet Gynecol.

[19] Yang M, Wang M, Li N (2024). Advances in pathogenesis of preeclampsia. Arch Gynecol Obstet.

[20] Vats H, Saxena R, Sachdeva MP, Walia GK, Gupta V (2021). Impact of maternal pre-pregnancy body mass index on maternal, fetal and neonatal adverse outcomes in the worldwide populations: a systematic review and meta-analysis. Obes Res Clin Pract.

[21] Barber E, Ram M, Mor L, Ganor Paz Y, Shmueli A, Bornstein S, Barda G, Schreiber L, Weiner E, Levy M (2023). Pregnancy and placental outcomes according to maternal BMI in women with preeclampsia: a retrospective cohort study. Arch Gynecol Obstet.

[22] Okby R, Harlev A, Sacks KN, Sergienko R, Sheiner E (2018). Preeclampsia acts differently in in vitro fertilization versus spontaneous twins. Arch Gynecol Obstet.

[23] Funaki S, Ogawa K, Ozawa N, Hosoya S, Okamoto A, Urayama KY, Morisaki N, Sago H (2023). Association between fetal sex and pregnancy outcomes among women with twin pregnancies: a multicenter cross-sectional study. Arch Gynecol Obstet.

[24] Brown RE, Noah AI, Hill AV, Taylor BD (2024). Fetal sexual dimorphism and preeclampsia among twin pregnancies. Hypertension.

[25] Kirkegaard I, Uldbjerg N, Oxvig C (2010). Biology of pregnancy-associated plasma protein-A in relation to prenatal diagnostics: an overview. Acta Obstet Gynecol Scand.

[26] Brosens I, Pijnenborg R, Vercruysse L, Romero R (2011). The, “Great Obstetrical Syndromes” are associated with disorders of deep placentation. Am J Obstet Gynecol.

[27] Wu P, van den Berg C, Alfirevic Z, O'Brien S, Röthlisberger M, Baker PN, Kenny LC, Kublickiene K, Duvekot JJ (2015). Early pregnancy biomarkers in pre-eclampsia: a systematic review and meta-analysis. Int J Mol Sci.

[28] Poon LC, Shennan A, Hyett JA (2019). The International Federation of Gynecology and Obstetrics (FIGO) initiative on pre-eclampsia: a pragmatic guide for first-trimester screening and prevention. Int J Gynaecol Obstet.

[29] Tan MY, Poon LC, Rolnik DL, Syngelaki A, de Paco MC, Nicolaides KH (2018). Prediction and prevention of small-for-gestational-age neonates: evidence from SPREE and ASPRE. Ultrasound Obstet Gynecol.

[30] ACOG Committee Opinion No (2018). 743: low-dose aspirin use during pregnancy. Obstet Gynecol.

[31] Hypertension in pregnancy: diagnosis and management. NICE Guideline, No. 133. London: National Institute for Health and Care Excellence (NICE). ISBN-13: 978-1-4731-3434-8. (2019). 31498578

[32] Toussia-Cohen S, Zaslavsky-Paltiel I (2023). Reconsidering the effectiveness of low-dose aspirin in prevention of pre-eclampsia among otherwise low risk twin gestations: a historical cohort study. Int J Gynecol Obstet.

[33] Bergeron TS, Roberge S, Carpentier C, Sibai B, McCaw-Binns A, Bujold E (2016). Prevention of preeclampsia with aspirin in multiple gestations: a systematic review and meta-analysis. Am J Perinatol.

[34] D’Antonio F, Khalil A, Rizzo G, Fichera A, Herrera M, Prefumo F (2023). Aspirin for prevention of preeclampsia and adverse perinatal outcome in twin pregnancies: a systematic review and meta-analysis. Am J Obstet Gynecol MFM.

